# Systematically analysing the acceptability of pig farming systems with different animal welfare levels when considering intra-sustainability trade-offs: Are citizens willing to compromise?

**DOI:** 10.1371/journal.pone.0282530

**Published:** 2023-03-08

**Authors:** Aurelia Schütz, Gesa Busch, Winnie Isabel Sonntag

**Affiliations:** 1 Department of Agricultural Economics and Rural Development, University of Goettingen, Goettingen, Lower Saxony, Germany; 2 Food Consumption and Wellbeing, University of Applied Sciences Weihenstephan-Triesdorf, Freising, Bavaria, Germany; University of Foggia: Universita degli Studi di Foggia, ITALY

## Abstract

In recent years, intensive pig husbandry has been subject to increasing public criticism, including a clear demand for more animal-friendly housing systems in many countries. However, such systems are associated with trade-offs at the expense of other sustainability domains, which challenges implementation and makes prioritization necessary. Overall, research is scarce that systematically analyses citizens’ evaluation of different pig housing systems and associated trade-offs. Given the ongoing transformation process of future livestock systems that meet social demands, it is crucial to include public attitudes. We therefore assessed how citizens evaluate different pig housing systems and whether they are willing to compromise animal welfare in trade-off situations. We conducted an online survey with 1,038 German citizens using quota and split sampling in a picture-based survey design. Participants were asked to evaluate several housing systems with different animal welfare levels and associated trade-offs based on an either positive (‘free-range’ in split 1) or negative (‘indoor housing with fully slatted floors’ in split 2) reference system. Initial acceptability was highest for the ‘free-range’ system, followed by ‘indoor housing with straw bedding and outdoor access’, ‘indoor housing with straw bedding’, and ‘indoor housing with fully slatted floors’, with only the latter being clearly not acceptable for many. Overall acceptability was higher with a positive rather than a negative reference system. When confronted with several trade-off situations, participants became uncertain and temporarily adjusted their evaluations. Thereby participants were most likely to trade off housing conditions against animal or human health rather than against climate protection or a lower product price. Nevertheless, a final evaluation demonstrated that participants did not fundamentally change their initial attitudes. Our findings provide evidence that citizens’ desire for good housing conditions is relatively stable, but they are willing to compromise at the expense of animal welfare up to a moderate level.

## Introduction

In recent years, livestock production has moved into the public focus in Western societies, with animal welfare turning out to be a focal point of interest and concern for many people. In this context, criticism towards farm animal welfare has been increasing, especially for highly intensive production systems such as poultry or pig husbandry [1–4]. In Germany, more than 90 percent of all pigs are kept in intensive production systems with extremely barren environments, where they are hardly able to display species-appropriate behaviour [5,6]. These housing systems are not in line with the societal idea of animal-friendly pig husbandry [7–9]. Accordingly, there is a clear public demand for more natural and species-appropriate housing systems, including sufficient space, outdoor access, natural floor conditions, enrichment material or mud wallows [7,9,10]. This is reflected by several studies that have analysed public perceptions and evaluations of different housing systems. Kühl et al. [8] systematically assessed the evaluation of pig housing systems with different levels of animal welfare and revealed that a free-range system was rated best, indoor housing with outdoor access second, outdoor climate housing third and indoor housing with slatted floors last. A study by Sonntag et al. [11] shows a clear rejection of farrowing crates for sows by most participants. Similarly, according to Busch et al. [12] and Wildraut et al. [10], pig housing systems solely equipped with slatted floors are not acceptable for many people.

However, animal-friendly husbandry systems are associated with numerous sustainability trade-offs, which challenges implementation. A trade-off can be defined as an, at least partial, incompatibility of two or more goals which need to be prioritized during decision making [13]. In the context of animal production, the most prominent trade-offs emerge between sustainability aspects such as animal welfare, environmental/climate protection or human health on the one hand and economic efficiency or consumer prices on the other hand [14]. Thus, e.g. an increase of stocking density leads to lower on-farm costs, but animal welfare suffers [15]. However, trade-offs can also occur between different sustainability aspects. While outdoor access, for example, improves animal welfare by providing different functional areas and climate zones and allows the expression of species-appropriate behaviour [16,17], gas and nutrient emissions with air or groundwater contamination can increase [16–18]. Furthermore, the increased exposure to parasites or contact with wildlife diseases in outdoor systems may pose animal and even human health at risk [16]. Similarly, straw used as enrichment material or bedding has several positive effects on animal welfare but is simultaneously accompanied with some health risk for pigs [16]. These trade-offs do not exclusively arise from a scientific but also from a societal point of view. Citizens are concerned not only about animal welfare but also about environmental protection and increasing consumer prices [19–21].

To support the ongoing transformation towards a more sustainable, future-oriented livestock production, it is indispensable to consider and carefully evaluate trade-offs that may arise. This also includes understanding how citizens evaluate individual advantages and disadvantages of different housing systems in a trade-off situation. However, up until now, only few studies have addressed this issue, with most of them rather produce and discuss the social perspective on trade-offs as a kind of secondary finding. Thus, for example, a study conducted by Winkel et al. [22], were participants had to evaluate the importance and feasibility of different animal welfare measures, demonstrates consumers’ poor awareness for possible challenges (e.g. trade-offs) associated with more animal friendly housing systems. In line with this, results show that when participants were asked about trade-offs in pig production, they had only a limited understanding and difficulties to name them. When they had existing knowledge, it was mainly limited to the conflict between price and animal welfare [9,11] for which consumers considered themselves to be responsible to some extent [23,24]. Trade-offs between different sustainability aspects such as emissions or animal welfare were not known by many. Wildraut et al. [10] found that the confrontation of citizens with trade-offs resulted in feelings of helplessness and guilt, from which they tried to escape by finding excuses for their own conflicting behaviour. Altogether, participants failed to succeed in solving trade-off situations [10] but in the end went for improved animal welfare in case of doubt [11,14,23,25]. Accordingly, using outdoor access and the farrowing crate as example, Sonntag et al. [11] showed that animal welfare arguments related to housing conditions (i.e. space, fresh air or daylight, expression of natural behaviour patterns or thermoregulation) outweighed arguments relating to economic, technological, hygiene or even animal health arguments. Furthermore, it turned out that information on advantages and disadvantages did not lead to a higher acceptability of a negatively perceived housing system nor to a lower acceptability of a positively perceived housing system [11]. Several willingness to pay studies underly consumers’ preference for animal welfare as they indicate consumers’ willingness to pay higher prices for products from more animal friendly production systems [26,27].

In a nutshell, there is increasing public desire for a change of pig production systems towards more animal-friendly housing conditions, with a clear preference for near-natural systems that are associated with high animal welfare levels. These preferences give rise to numerous trade-offs that need to be considered in the design of future, more sustainable livestock systems. Analysing to what extent citizens are willing to compromise at the expense of animal welfare by accepting housing systems that may not correspond to their ideal concept, but show benefits for other sustainability dimensions, is important for valuing sustainability in future animal farming. To address this issue, we chose a picture-based study design with German residents. In total, participants had to evaluate four housing systems with different animal welfare levels and associated trade-offs. For this, they were assigned to one of two split samples and either received a positive (‘free-range’ in split 1) or a negative (‘indoor housing with fully slatted floors’ in split 2) housing system as a reference for their evaluation. Using this approach, we aimed to investigate citizens’ dealing with trade-offs from different perspectives and to test whether their evaluation was subject to cognitive biases. To the best of our knowledge, this is the first study which systematically analyses citizens’ willingness to trade-off animal welfare (i.e. housing conditions) against a number of other sustainability aspects (e.g. environmental and climate protection or protection of human health) using several housing systems with different animal welfare levels.

Thus, the following research questions were addressed in our study:

1) How do citizens accept pig housing systems with different levels of animal welfare?
  1a) How do citizens evaluate the two reference systems ‘free-range’ and ‘indoor housing with fully slatted floors’?
  1b) How do citizens accept several housing systems presented as alternatives to the reference systems?
  1c) Is acceptability of the alternative housing systems influenced by the type of reference system (i.e. positive or negative system)?
2) Are citizens willing to compromise on animal welfare levels in sustainability trade-off situations by accepting alternative housing systems?
  2a) Does citizens’ willingness to compromise depend on the specific trade-off situation?
  2b) Does citizens’ willingness to compromise change through confrontation with trade-off situations (i.e. temporarily and sustainably)?

## Materials and methods

### Study design

**Fig 1:**
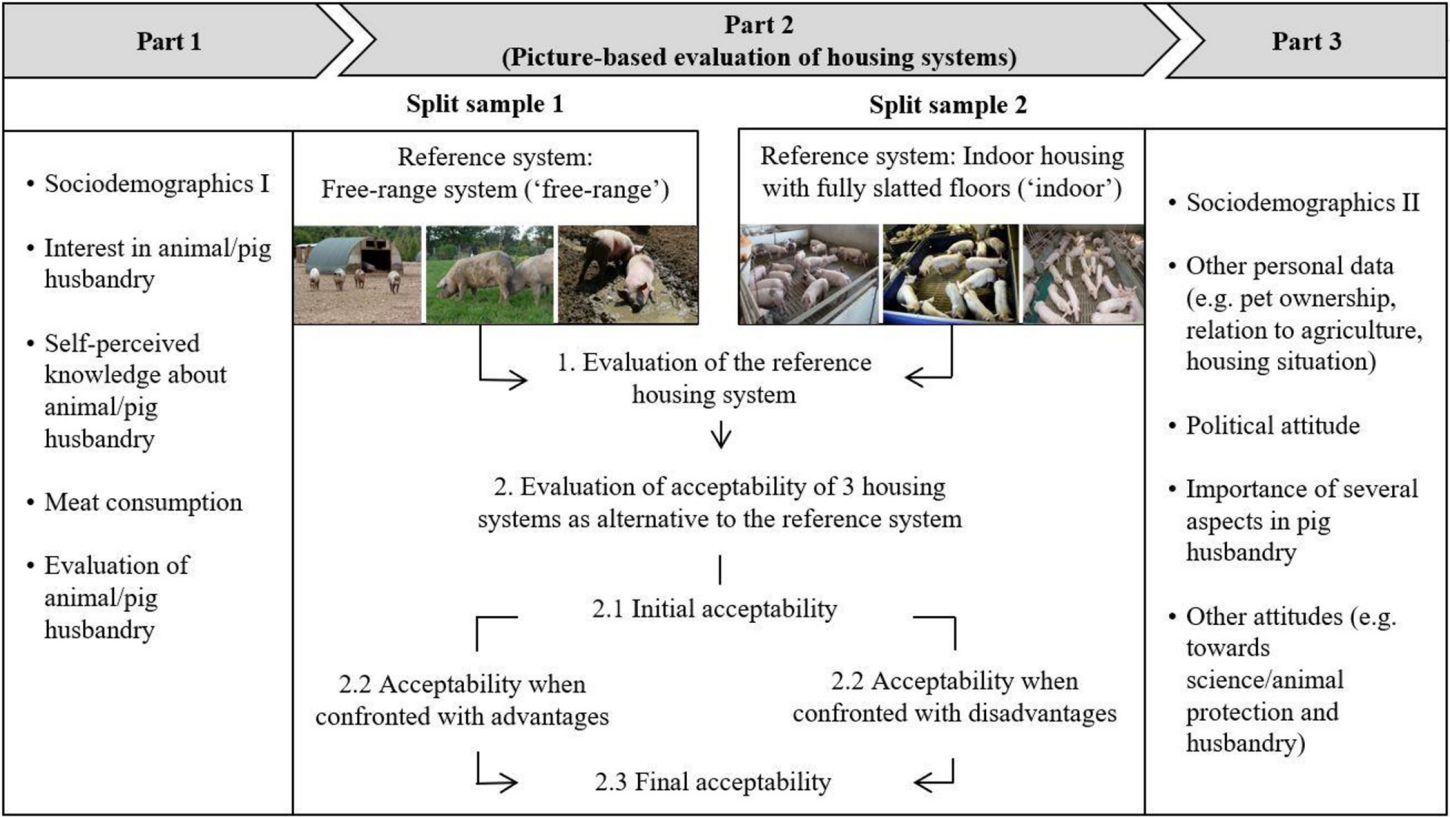
Overview of the study design (structure of questionnaire).

Data was collected using a standardized online questionnaire with a cross-sectional design as we considered it to be the most suitable approach for the purpose of our study. Thus, we were e.g. able to obtain a large sample whose composition corresponds to the German population in terms of some sociodemographic characteristics. We further were able to use a picture-based approach and split sampling design, to randomize questions and items to avoid order effects and to benefit from easy data entry and analysis while at the same time keeping efforts and costs at a reasonable level [28–31]. The questionnaire consisted of three parts (Fig 1). While parts 1 and 3 of the questionnaire were the same for all participants, for the picture-based part 2, participants were randomly assigned to one out of two split samples. We aimed to investigate citizens’ general acceptability of different housing systems as well as their dealing with trade-offs. Using split sampling, we were able to include the latter from two different perspectives as different reference housing systems were introduced between the groups (for a more detailed description see section ‘part 2 of the questionnaire’). We opted for a picture-based presentation of the housing systems accompanied by a brief description to ensure common understanding. To provide realistic pictures, we sent the pictures and descriptions to an animal welfare organization and a farmers’ association before data collection. Both confirmed that our selection represented realistic scenarios of pig farming. However, with regard to the housing system ‘indoor housing with fully slatted floors’, the animal welfare organization mentioned that the pictures represent a rather positive example of a conventional pig stable due to a comparatively high level of daylight. Additionally, we conducted cognitive pre-tests with non-experts (n = 11) as well as experts from our working group ‘consumer behaviour’ (n = 8) in order to ensure overall comprehensibility and length of the questionnaire as well as perception of the selected housing systems. The results show that some non-expert pre-testers considered the ‘indoor housing with fully slatted floors’ a comparatively good housing system since they had expected even worse systems. This should be considered when interpreting the results.

### Structure of the questionnaire

#### Parts 1 and 3 of the questionnaire

Part 1 included questions about sociodemographic characteristics, self-perceived knowledge about and interest in animal and pig husbandry, meat and meat product consumption and the acceptance and evaluation of animal and pig husbandry. Part 3 consisted of further questions about sociodemographic characteristics, other personal data (e.g. pet ownership and relation to agriculture), political attitudes, importance of several aspects in pig husbandry, as well as other attitudes (e.g. towards science, animal protection and animal husbandry).

#### Part 2 of the questionnaire (picture-based evaluation of housing systems)

**Fig 2:**
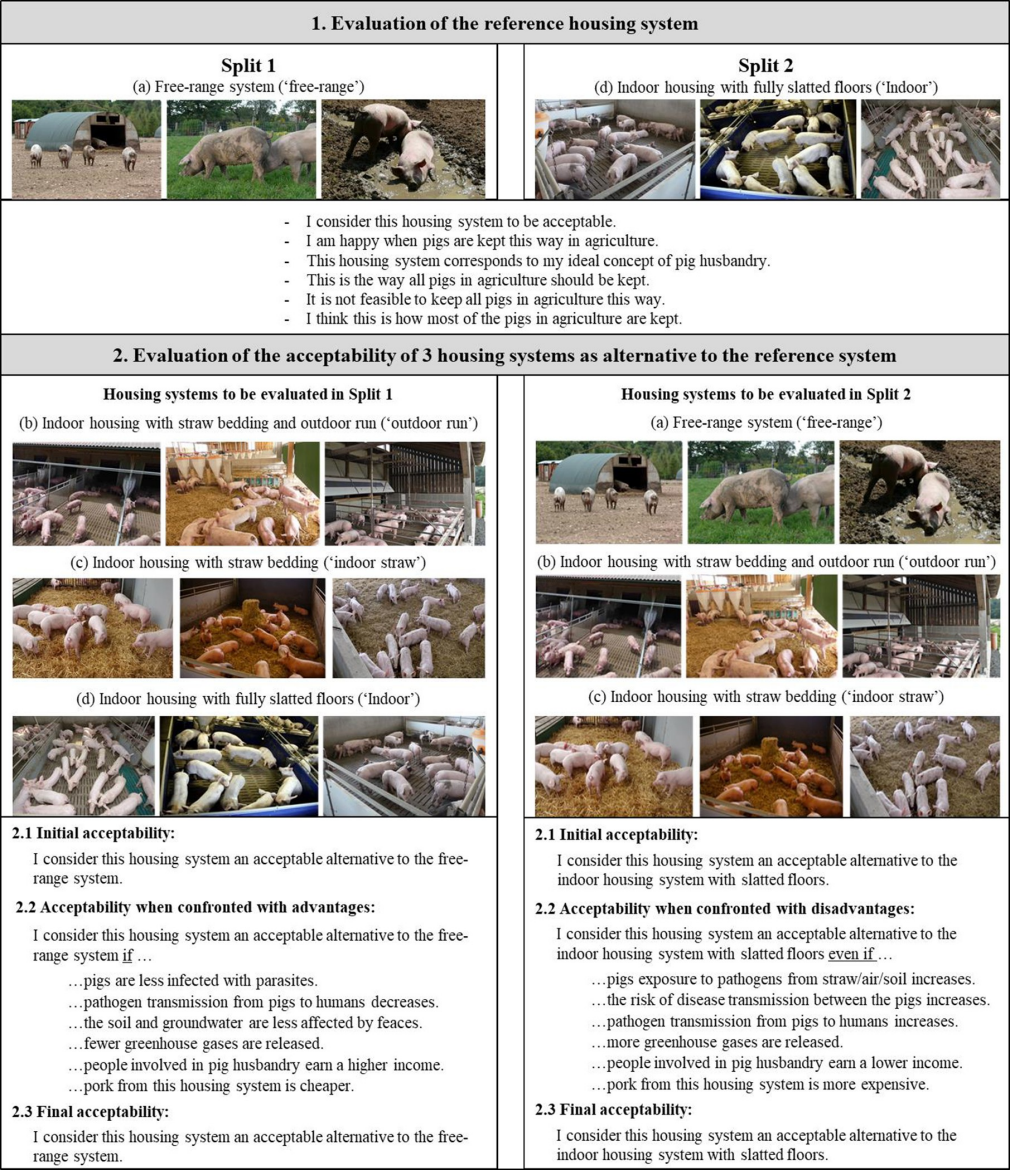
Structure of the picture-based part of the questionnaire (part 2). The three housing systems to be evaluated as alternatives to the reference system in each split were presented in randomized order. Evaluation of the reference system and the acceptability of the three alternative housing systems on a 7-point Likert scale from 1 = completely disagree, 4 = partly/partly, 7 = totally agree. Source: a) Free-range: Bildagentur Landpixel (1 & 2), Verein Happy Pigs & Friends; b) Outdoor run: ÖKL-Baupreis 2012, www.oekl-bauen.at, Rudolf Wiedmann, Stephan Fritzsche, KTBL; c) Indoor straw: DVS, Ella Martin, Bildagentur Landpixel; d) Indoor: Bildagentur Landpixel, Jana Denecke, Tierhaltung modern und transparent e.V.

Fig 2 shows an overview of the picture-based part 2 of the questionnaire, including questions that were asked.

In both split samples participants had to evaluate four different pig housing systems and associated trade-offs (i.e. advantages and disadvantages), each based on three pictures and brief descriptions. Thereby, one housing system served as a reference system that was presented first. The remaining three were evaluated as possible alternatives to this reference system. The four housing systems and associated descriptions were: (a) ‘Free-range system’: The pigs live all year round outside on pasture with a shelter (huts or similar); (b) ‘Indoor housing with straw bedding and outdoor run’: The pigs are kept in a barn with straw and have year-round access to an outdoor run with slatted floors; (c) ‘Indoor housing with straw bedding’: Pigs are kept in a barn with straw all year round and do not have access to an outdoor run, and (d) ‘Indoor housing with fully slatted floors’: Pigs are kept in a barn all year round with slatted floors and have no access to an outdoor run. For simplicity reasons we will use the abbreviations (a) ‘free-range’, (b) ‘outdoor run’, (c) ‘indoor straw’ and (d) ‘indoor’ hereafter.

Participants in each split sample saw pictures from the dedicated reference system first, namely ‘free-range’ in split 1 and ‘indoor’ in split 2. From these two systems we already know that they are perceived and evaluated very differently by citizens [8,10,12]. Accordingly, split 1 started with a positively (‘free-range’) and split 2 with a negatively (‘indoor’) connotated reference system. In the next step, all participants had to indicate their acceptability of three other housing systems as alternatives to the respective reference system (hereafter referred to as alternative housing systems). Thereby, questions were asked both in general as well as with regard to specific trade-offs. The general evaluation had to be made twice, once before and once after evaluating the trade-offs (i.e. initial and final acceptability see Fig 2). Trade-offs were introduced as advantages of the alternative housing systems over the reference system in split 1 (‘free-range’) and as disadvantages in split 2 (‘indoor’). Alternative housing systems and trade-offs were presented and evaluated in randomized order.

### Selection of housing systems and associated trade-offs used in the study

For our study, we selected four different pig housing systems (Fig 2), all of which are actually used in practice and are associated with trade-offs (i.e. advantages and disadvantages) of varying intensities with regard to animal welfare and other sustainability as well as economic aspects. Especially when it comes to housing conditions, the systems clearly differ in terms of their environmental enrichment levels and therewith in their benefits for the animals. The term environmental enrichment implies measures that aim at adjusting the environment of captive animals in a way that improves animal welfare [32–34], including diverse methods such as physical environment, social companionship or nutritional delivery and type [35]. In this context for split 1, we chose the housing system with the highest enrichment level as a reference system (i.e. ‘free-range’), whereas for split 2 we chose the system with the lowest enrichment level (i.e. ‘indoor’). This means that in split 1, housing systems presented as alternatives were all worse regarding housing conditions compared with the reference system ‘free-range’. Similarly, in split 2, the presented alternatives were all better than the reference system ‘indoor’. Accordingly, participants from split 1 were confronted with several advantages related to animal health, human health, environmental and climate impact, the pig farmer’s income and product price (for a detailed list of all presented advantages see Fig 2), and participants from split 2 faced several disadvantages of the same aspects.

These trade-offs (advantages and disadvantages) were selected based on existing literature as well as expert discussions carried out by one of our partners in the project ‘SocialLab II–Acceptance through Innovation’ within the framework of another work package. Finally, we chose advantages/disadvantages that are relevant in pig farming, cover different sustainability and economic aspects and can be applied to all selected housing systems in both splits.

### Sample

Participants were recruited by an online panel provider in January 2021. Subjects were selected using quota sampling with gender, age, place of residence (North, South, East, and West Germany) and household income as quota control criteria according to the distribution in the German population (Table 1). Out of 1,109 respondents who completed the questionnaire, 71 were deleted from the final dataset due to inappropriate response behaviour. Consequently, 65 participants were removed because they were identified as speeders (i.e. response time was less than half of the median response time of the overall sample: 8.5 minutes), and six were classified as straightliners (i.e. no variance in response behaviour). Both elimination procedures are commonly used to improve data quality in online surveys [36]. The final sample size comprised 1,038 respondents.

**Table 1:** Sociodemographic characteristics of the total sample, sample splits and German population.

Specification		Total sample (%)	Split 1 (%)	Split 2 (%)	German population (%)
**Gender**					
	Male	47.9	48.8	46.9	49.1
	Female	51.7	50.6	52.9	50.9
	Other	0.4	0.6	0.2	—
**Age in years**					
	18–29	15.6	14.4	16.8	16.3
	30–39	14.2	14.2	14.1	15.5
	40–49	14.2	14.2	14.1	14.7
	50–59	19.5	20.2	18.7	19.4
	60 and older	36.6	36.9	36.3	34.1
**Place of residence**					
	South^1^	28.7	29.8	27.6	29.1
	North^2^	16.0	15.8	16.2	16.1
	East^3^	19.4	19.8	18.9	19.5
	West^4^	35.9	34.6	37.3	35.3
**Household income (EUR)**					
** **	Under 1300	17.9	17.5	18.3	18.4
	1300–2599	36.5	36.7	36.3	36.6
	2600–4499	28.9	28.3	29.5	28.7
	4500 and more	16.7	17.5	15.8	16.3

Total sample: n = 1.038; split 1: n = 520; split 2: n = 518. Statistisches Bundesamt Germany 2019.

^1^ Bavaria, Baden-Wuerttemberg.

^2^ Bremen, Hamburg, Lower Saxony, Schleswig-Holstein.

^3^ Brandenburg, Berlin, Saxony, Saxony-Anhalt, Thuringa, Mecklenburg-Western Pomerania.

^4^ Hesse, North Rhine-Westphalia, Rhineland-Palatinate, Saarland.

Cross-tabulation with χ^2^-tests were used to test for differences between split 1 and split 2; no differences were found for none of the sociodemographic characteristics (p > 0.05). The gender category ‘other’ was not included in the analysis because of the small number of cases (n = 4). Gender: χ^2^ (1) = 0.469; age: χ^2^ (4) = 1.258; place of residence: χ^2^ (3) = 1.081, df = 3; income: χ^2^ (3) = 0.694.

### Ethic statement

This study was conducted in accordance with the Ethical Principles of the German Psycho-logical Society (DGP) and the Association of German Professional Psychologists (BDP). At the time of data collection, it was not mandatory at Goettingen University to obtain ethical approval for surveys related to attitudes such as the present study. The survey has been designed in a way that it would not cause distress or have any negative impact on the participants. The confidentiality of collected data was guaranteed by the online access panel provider which was in charge of recruiting participants in accordance with the general data protection regulation (GDPR) and the legal guidelines of the European Society of Opinion and Marketing Research (ESOMAR), the German Society for Online Research (DGOF), as well as the Association of German Market and Social Research Institutes e. V. (ADM). Furthermore, data collection, processing and usage of personal data were carried out in full accordance with the strict guidelines of the German Bundesdatenschutzgesetz (BDSG). Before starting the survey, participants were asked to consent their voluntary participation in the study by clicking on a corresponding button. All participants were informed that they had the right to withdraw from the study at any time without giving any reasons by closing their internet browser. The study used only anonymous questionnaires. All participants were advised that data will be analysed anonymously and cannot be identified and/or linked to individual participants.

### Statistical analysis

Data were analysed using IBM SPSS Statistics 26. For descriptive purposes we used mean scores, standard deviations and frequencies. For bivariate analysis we used a t-test for dependent samples to compare means within a split sample, and a t-test for independent samples to compare means between split samples. To test for differences between split samples regarding sociodemographic characteristics we used cross-tabulation with χ ^2^-test. All tests were two-tailed, and the significance level was set at 0.05.

## Results

### Sample description

Table 1 shows the distribution of sociodemographic characteristics set as quota control criteria in the total sample, the split samples as well as the German population.

There was only little deviation from the German population in both splits and cross-tabulation with χ 2-tests revealed no differences between split samples (p > 0.05). Similarly, cross-tabulation with χ2-tests showed no differences in dietary behaviour between splits (χ^2^ (4) = 6.607, p > 0.05): most of the participants in both splits are meat eaters (split 1 = 93.7%; split 2 = 92.7%) and, accordingly, only few follow a pescetarian, vegetarian or vegan diet (split 1 = 6.3%; split 2 = 7.3%). Among meat eaters, 40.6% consume little (i.e. rather little, little, very little), 38.5% average amount and 14.6% much meat (i.e. rather much, much, very much) in split 1 and 38.0% consume little, 35.1% average amount and 19.5% much meat in split 2. With regard to further characteristics, such as evaluation or involvement in animal and pig husbandry, comparison of means showed that both splits were also quite similar (p > 0.05). Thus, on average, participants from both splits rated current German animal husbandry in general as well as pig husbandry in particular as rather unacceptable. Similarly, pig welfare on farms was rated rather poorly. Furthermore, on average, participants indicated a moderate interest in as well as knowledge about animal and pig husbandry (Table 2).

**Table 2:** Evaluation of and involvement in animal and pig husbandry for the total sample and the split samples 1 and 2.

	Mean (SD)		
	Total sample (n = 1,038)	Split 1 (n = 520)	Split 2 (n = 518)
Animal husbandry is acceptable^1^	3.42 (1.50)	3.38 (1.48)	3.46 (1.53)
Pig husbandry is acceptable^1^	3.20 (1.52)	3.13 (1.49)	3.28 (1.54)
Rating of pig welfare^2^	3.06 (1.30)	3.06 (1.29)	3.07 (1.30)
Interest in animal husbandry^3^	4.45 (1.50)	4.49 (1.51)	4.41 (1.50)
Interest in pig husbandry^3^	4.39 (1.55)	4.47 (1.55)	4.30 (1.54)
Knowledge about animal husbandry^3^	3.72 (1.31)	3.72 (1.30)	3.72 (1.32)
Knowledge about pig husbandry^3^	3.62 (1.32)	3.62 (1.31)	3.62 (1.34)

Displayed are means and standard deviations (SD) in brackets. Rating on 7-point Likert scales: ^1^from 1 = completely disagree, 4 = partly/partly, 7 = totally agree; ^2^from 1 = very bad, 4 = partly/partly, 7 = very good; ^3^from 1 = very low, 4 = moderate, 7 = very high. Comparison of means between splits using t-test for independent samples: We found no differences for none of the statements (p > 0.05).

### Evaluation of the two reference systems ‘free-range’ and ‘indoor’ (research question 1a)

On average, participants from split 1 rated their reference housing system ‘free-range’ positively, whereas participants from split 2 rated their reference system ‘indoor’ negatively (Table 3). For example, participants from split 1 strongly agreed with the statement ‘I consider this housing system to be acceptable’ (μ = 6.31, σ = 0.99) and ‘This housing system corresponds to my ideal image of pig husbandry’ (μ = 6.08, σ = 1.17), whereas agreement in split 2 was low for these statements (μ = 2.11; σ = 1.31 and μ = 1.76; σ = 1.21). Furthermore, participants belonging to split 1 did not believe that most of the pigs in Germany are kept in ‘free-range’ systems (μ = 2.10, σ = 1.31). However, this was much more likely for the ‘indoor’ housing in split 2, even though there was only moderate agreement (μ = 4.71, σ = 1.34) (Table 3).

**Table 3:** Evaluation of the two reference housing systems in splits 1 and 2.

	‘Free-range’ (Split 1) (n = 520)	‘Indoor’ (Split 2) (n = 518)
I consider this housing system to be acceptable.	6.31 (0.99)	2.11 (1.31)
I am happy when pigs are kept this way in agriculture.	6.28 (1.04)	1.93 (1.32)
This housing system corresponds to my ideal concept of pig husbandry.	6.08 (1.17)	1.76 (1.21)
This is the way all pigs in agriculture should be kept.	5.91 (1.19)	1.91 (1.29)
It is not feasible to keep all pigs in agriculture this way.	4.11 (1.76)	3.30 (1.70)
I think this is how most of the pigs in agriculture are kept.	2.10 (1.31)	4.71 (1.34)

Displayed are means and standard deviations in brackets.

Rating on a 7-point Likert scale from 1 = completely disagree, 4 = partly/partly, 7 = totally agree. Comparison of means between splits using t-test for independent samples: Differences were significant for all statements (p ≤ 0.001) but are not marked in the table.

### Acceptability of alternative housing systems and influence of the reference system (research questions 1b & 1c)

**Fig 3:**
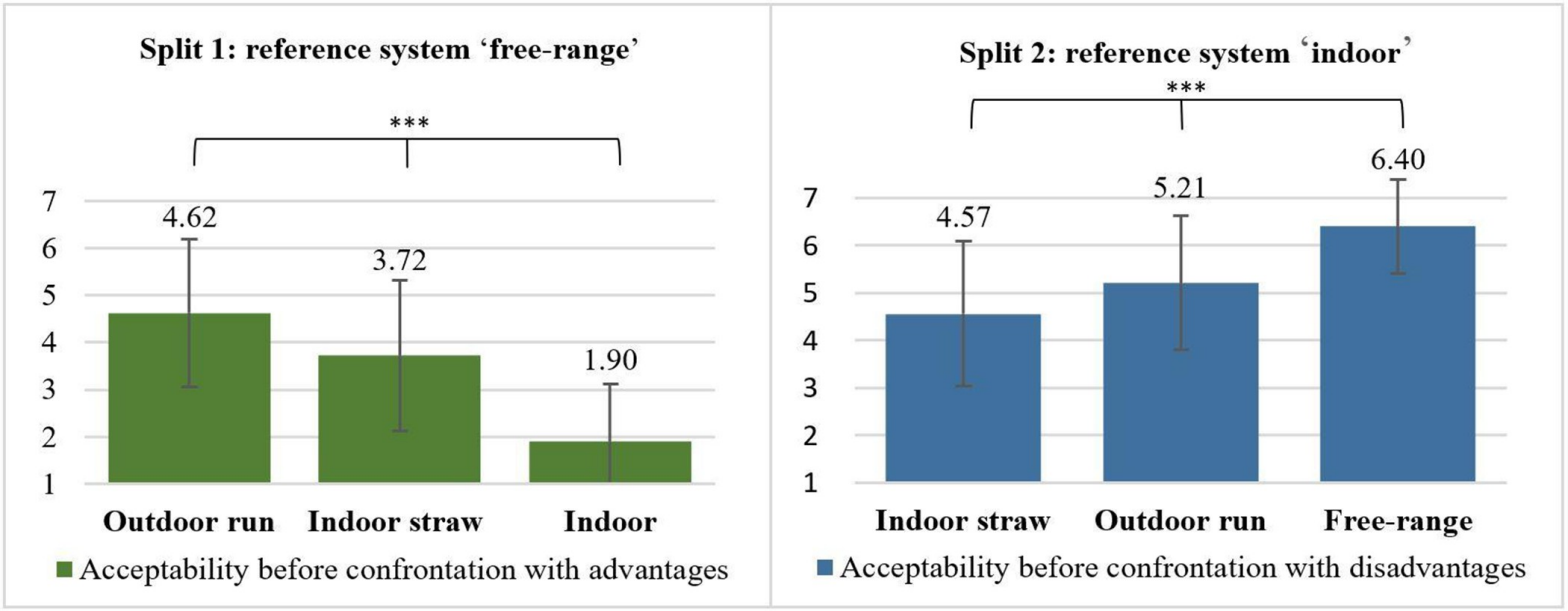
Initial acceptability of alternative housing systems (before confrontation with trade-offs). Split 1: n = 520; split 2: n = 518. Displayed are means and standard deviations. Split 1: Rating of the statement ‘I consider this housing system an acceptable alternative to the free-range system’. Split 2: ‘I consider this housing system an acceptable alternative to the indoor housing system with slatted floors’. Rating on a 7-point Likert scale from 1 = completely disagree, 4 = partly/partly, 7 = totally agree. Comparison of acceptability levels between housing systems within a split sample using t-test for dependent samples; significant differences with *** p ≤ 0.001. Comparison of acceptability of ‘indoor straw’ and ‘outdoor run’ between split samples using t-test for independent samples; differences were significant (p ≤ 0.001) but are not marked in the figure.

Participants from split 1 were asked to rate the statement ‘I consider this housing system an acceptable alternative to the free-range system’ before they were confronted with different trade-off situations (i.e. advantages) (Fig 2). Firstly, comparison of means revealed that initial acceptability differed between all three alternative housing systems (p ≤ 0.001), with the highest acceptability for ‘outdoor run’, followed by ‘indoor straw’ and ‘indoor’ (Fig 3).

Accordingly, participants from split 2 were asked to rate the statement ‘I consider this housing system an acceptable alternative to the indoor housing system with slatted floors’ for each alternative housing system, before they were confronted with different trade-off situations (i.e. disadvantages) (Fig 2). Comparison of means showed that acceptability was different between all three alternative housing systems (p ≤ 0.001), with highest acceptability for the housing system ‘free-range’, followed by ‘outdoor run’ and ‘indoor straw’ (Fig 3).

Further, comparison of means revealed differences between the two split samples for ‘indoor straw’ and ‘outdoor run’ (p ≤ 0.001), which were the only housing systems included as alternatives in both splits and can thus be compared. Acceptability levels in split 2 were higher compared to split 1 for both housing systems (‘indoor straw’: split 1: μ = 3.72, σ = 1.60, split 2: μ = 4.57, σ = 1.53; ‘outdoor run’: split 1: μ = 4.62, σ = 1.57, split 2: μ = 5.21, σ = 1.41) (see Fig 3).

### Willingness to compromise depending on the trade-off situation (research question 2a)

**Fig 4:**
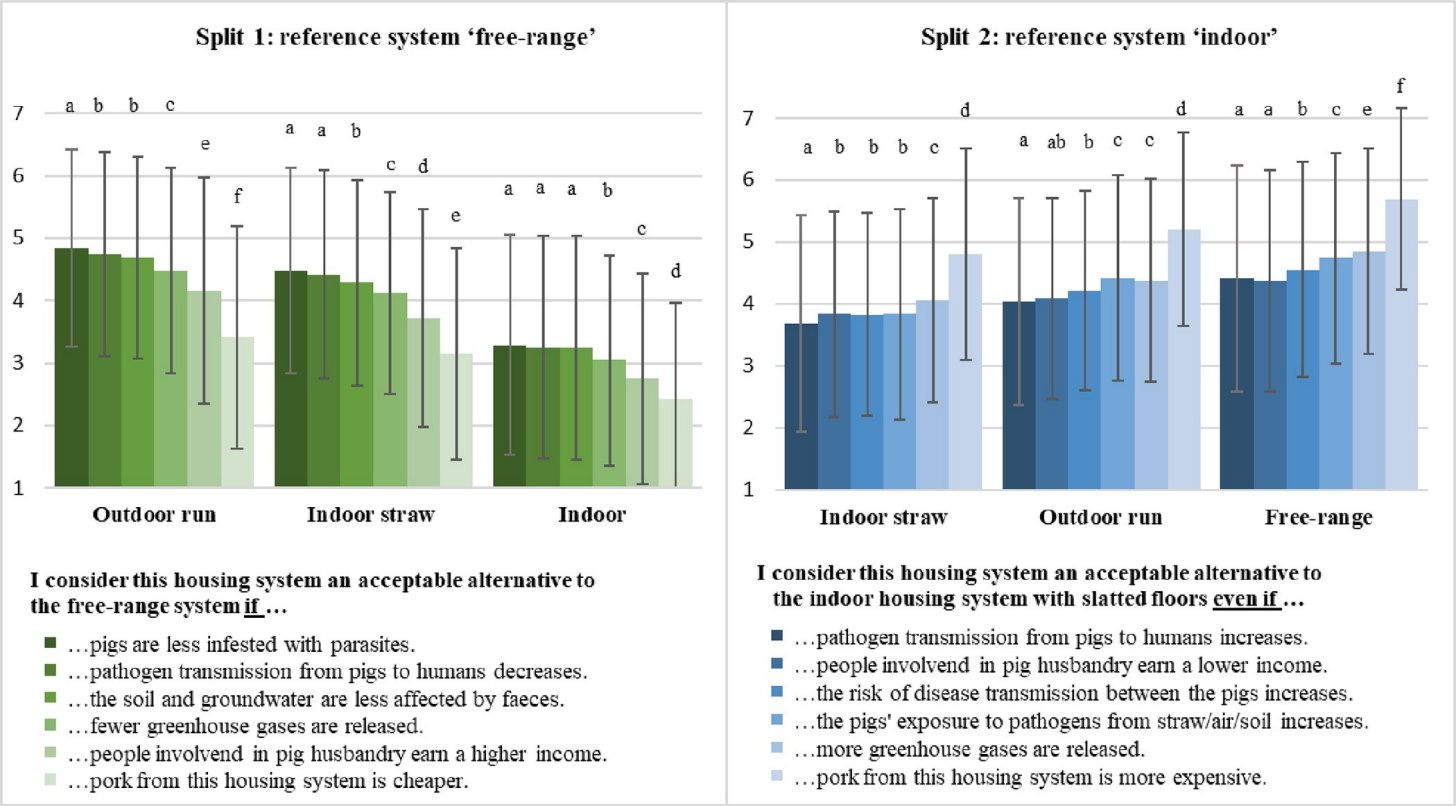
Acceptability of alternative housing systems when confronted with different trade-off situations. Split 1: n = 520; split 2: n = 518; displayed are means and standard deviations. Rating on a 7-point Likert scale from 1 = completely disagree, to 4 = partly/partly, to 7 = totally agree. Comparison of means within a split sample using t-test for dependent samples. Different letters indicate significant differences (p ≤ 0.05) between advantages/disadvantages within a housing system, and same letters indicate no significant differences (p > 0.05). Comparison of means between housing systems revealed significant differences between the same advantages/disadvantages (p ≤ 0.001) but are not marked in the figure.

Further, participants were asked to evaluate their acceptability of alternative housing systems in the context of different trade-off situations (i.e. advantages/disadvantages) to show whether willingness to compromise depends on the specific advantages/disadvantages presented. When confronted with several advantages of alternative housing systems compared to the reference system ‘free-range’ (split 1), participants’ average acceptability was highest for all given advantages in the case of ‘outdoor run’ and lowest in the case of ‘indoor’ (differences between housing systems with p ≤ 0.001) (Fig 4). Looking at the acceptability when confronted with disadvantages (split 2), we found differences between all alternative housing systems, similar to split 1 (p ≤ 0.001). On average, disadvantages received the lowest acceptability for ‘indoor housing with straw bedding’ and the highest acceptability for ‘free-range’ (Fig 4).

The order in which advantages (split 1) received agreement was the same for all alternative housing systems, with the highest agreement for the advantage of a reduced infection with parasites, and the lowest for the advantage of a cheaper product price (Fig 4). However, comparison of means revealed either differences on a rather low level or no differences at all for most of the advantages, with product price being an exception. Furthermore, differences did not exist between the same advantages for all housing systems (Fig 4). For example, differences between the advantage of a reduced pathogen transmission from pigs to humans and the advantage of a reduced impact on soil and groundwater were not existent in the case of ‘outdoor run’ and ‘indoor’ and only on a low level for ‘indoor straw’.

The order of agreement levels when confronted with disadvantages (split 2) was different and more inconsistent than in split 1. Thus, instead of disadvantages related to animal health, disadvantages referring to other aspects such as pathogen transmission to humans or income of people involved in pig husbandry were more convincing (Fig 4). However, the disadvantage referring to a higher product price received the highest agreement for all housing systems and therefore had the weakest persuasive power–similar to split 1 (Fig 4). Also similar to split 1, a comparison of means shows that there were no differences between all disadvantages or were on a very low level in many cases. Furthermore, there were no differences between the same disadvantages for all housing systems.

### Changes in citizens’ willingness to compromise through confrontation with different trade-off situations (research question 2b)

First, we compared the initial acceptability of the alternative housing systems with the acceptability levels when confronted with the different trade-off situations (i.e. advantages/disadvantages) to test whether acceptability changed temporarily. Comparison of means revealed a higher acceptability for most of the evaluated advantages (split 1) compared to the initial acceptability (p ≤ 0.05). This effect depended on the housing system and became higher with decreasing animal welfare levels (i.e. strongest effect for ‘indoor’) (Table 4).

**Table 4:** Comparison of initial acceptability of alternative housing systems with acceptability levels when confronted with different trade-off situations.

Split sample 1 (n = 520)			
	Outdoor run	Indoor straw	Indoor
**Initial acceptability of the alternative housing systems**	4.62 (1.57)	3.72 (1.60)	1.90 (1.22)
**Acceptability when confronted with advantages**		** **	** **
I consider this housing system an acceptable alternative to the free-range system if …	** **		
… pigs are less infested with parasites.	4.84 (1.59) ***	4.48 (1.65) ***	3.29 (1.77) ***
… pathogen transmission from pigs to humans decreases.	4.75 (1.63) *	4.42 (1.67) ***	3.25 (1.79) ***
… the soil and groundwater are less affected by faeces.	4.69 (1.62)	4.29 (1.65) ***	3.25 (1.79) ***
… fewer greenhouse gases are released.	4.48 (1.65) *	4.13 (1.62) ***	3.05 (1.69) ***
… people involved in pig husbandry earn a higher income.	4.16 1.82) ***	3.72 (1.74)	2.75 (1.69) ***
… pork from this housing system is cheaper.	3.41 (1.79) ***	3.15 (1.70) ***	2.43 (1.54) ***
**Split sample 2 (n = 518)**			
	**Indoor straw**	**Outdoor run**	**Free-range**
**Initial acceptability of the alternative housing systems**	4.57 (1.53)	5.21 (1.41)	6.40 (0.99)
**Acceptability when confronted with disadvantages**	** **		
I consider this housing system an acceptable alternative to the indoor housing system with slatted floors even if …			
… pathogen transmission from pigs to humans increases.	3.69 (1.74) ***	4.04 (1.67) ***	4.41 (1.82) ***
… people involved in pig husbandry earn a lower income.	3.83 (1.66) ***	4.09 (1.62) ***	4.37 (1.78) ***
… the risk of disease transmission between the pigs increases.	3.83 (1.65) ***	4.21 (1.60) ***	4.55 (1.74) ***
… the pigs’ exposure to pathogens from straw/air/soil increases.	3.83 (1.70) ***	4.41 (1.66) ***	4.74 (1.70) ***
… more greenhouse gases are released.	4.05 (1.65) ***	4.37 (1.63) ***	4.84 (1.66) ***
… pork from this housing system is more expensive.	4.81 (1.70) **	5.20 (1.56)	5.69 (1.46) ***

Displayed are means and standard deviations. Ratings on a 7-point Likert scale ranged from 1 = completely disagree, to 4 = partly/partly, to 7 = totally agree. Comparison of means within a housing system using a t-test for dependent samples. Asterisks mark significant differences between the initial acceptability of a housing system and the acceptability when confronted with advantages/disadvantages.

However, the agreement levels when confronted with advantages of fewer soil and groundwater impacts stayed equal or were even lower compared to advantages referring to lower greenhouse gas emissions, a higher income of people working in pig husbandry and a cheaper product price in the case of ‘outdoor run’ (p ≤ 0.05). Furthermore, for ‘indoor straw’, the agreement for the advantage referring to a higher income stayed equal and was even lower for cheaper prices (p ≤ 0.001) (Table 4).

Comparing acceptability levels when confronted with disadvantages (split 2) with the initial acceptability levels, it turned out that acceptability was lower for most of the evaluated disadvantages (p ≤ 0.001). This effect varied depending on the housing system but became more important with increasing animal welfare levels (i.e. strongest effect for ‘free-range’). However, the acceptability of the disadvantage referring to a higher price stayed equal for ‘outdoor run’ and was even higher for the housing system ‘indoor straw’ (p ≤ 0.01) (Table 4).

**Fig 5:**
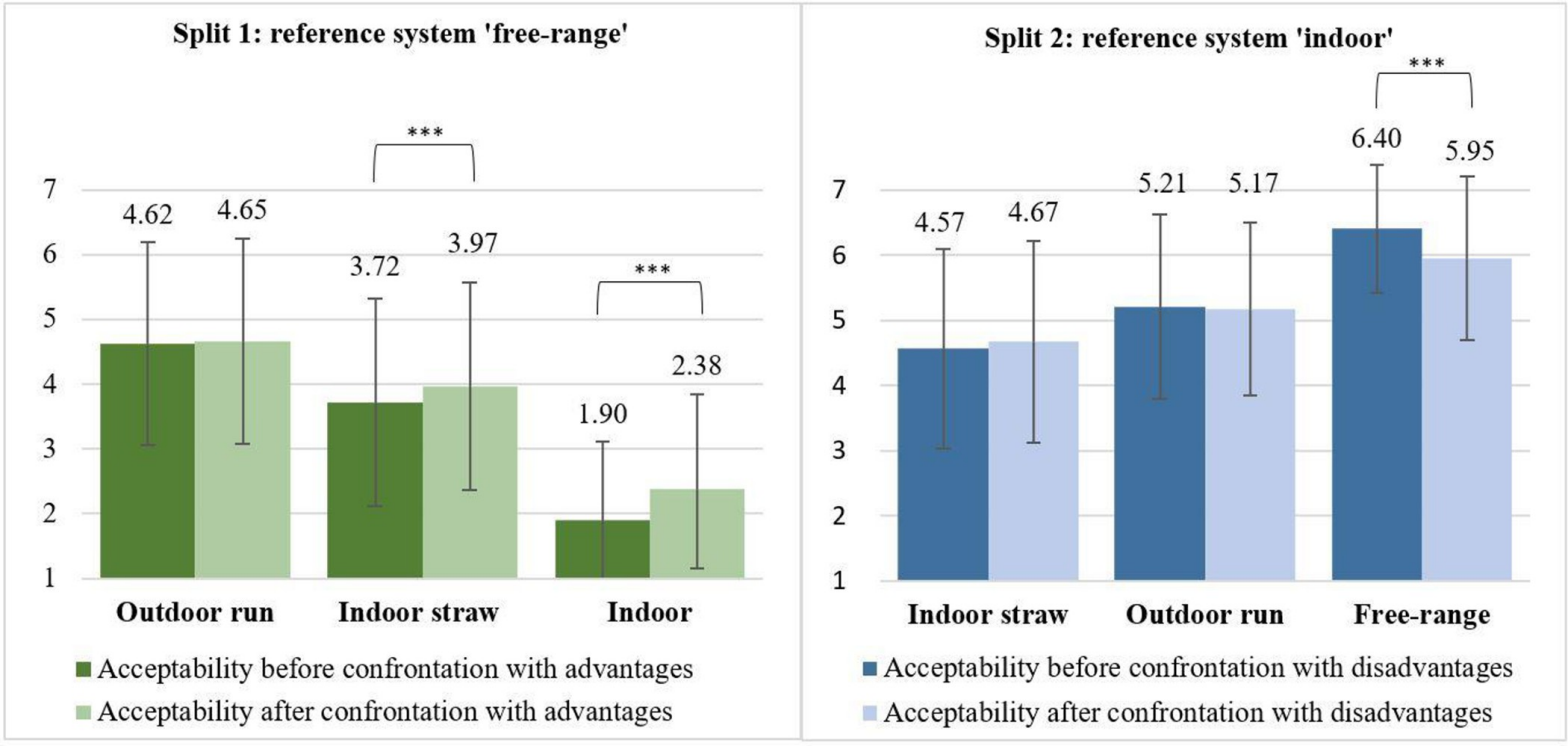
Comparison of acceptability levels of alternative housing systems before and after confrontation with trade-offs. Split 1: n = 520; split 2: n = 518. Displayed are means and standard deviations. Split 1: Rating of the statement ‘I consider this housing system an acceptable alternative to the free-range system’. Split 2: ‘I consider this housing system an acceptable alternative to the indoor housing system with slatted floors’. Ratings on a 7-point Likert scale ranged from 1 = completely disagree, to 4 = partly/partly, to 7 = totally agree. Comparison of means between acceptability before and after the confrontation with advantages/disadvantages within a housing system and split sample using a t-test for dependent samples; significant differences with *** p ≤ 0.001.

Secondly, we compared acceptability levels for the alternative housing systems before and after confrontation with trade-off situations (i.e. initial vs final acceptability, see Fig 2) to test whether acceptability changed not only temporarily (i.e. at the moment of confrontation with trade-offs) but also sustainably. In split 1, we found an increase in the acceptability of ‘indoor’ (before: μ = 1.90; σ = 1.22; after: μ = 2.38; σ = 1.46) and ‘indoor straw’ (before: μ = 3.72; σ = 1.60; after: μ = 3.97; σ = 1.59) (p ≤ 0.001) but not for ‘outdoor run’ (before: μ = 4.62; σ = 1.57; after: μ = 4.65; σ = 1.60) (p > 0.05) (Fig 5). Fig 5 shows that in split 2, the comparison of means between the acceptability before and after confrontation with trade-offs revealed no differences, neither for ‘indoor straw’ (before: μ = 4.57; σ = 1.53; after: μ = 4.67; σ = 1.55) nor for ‘outdoor run’ (before: μ = 5.21; σ = 1.41; after: μ = 5.17; σ = 1.33) (p > 0.05). In contrast, acceptability for ‘free-range’ decreased after disadvantages were introduced (before: μ = 6.40; σ = 0.99; after: μ = 5.94; σ = 1.26) (p ≤ 0.001).

## Discussion

### Evaluation of presented housing systems (research question 1)

Our first research question aimed to find out how citizens evaluate the acceptability of different pig housing, including a reference system (1a) and alternative housing systems (1b), and whether the type of reference system influenced their evaluation (1c). Our results show that participants evaluated the reference system ‘free-range’ positively, whereas the reference system ‘indoor’ (i.e. with fully slatted floors) was evaluated poorly. This is in line with previous studies demonstrating the prevailing aversion to intensive pig production systems and the general preference by large parts of the society for more natural and species-appropriate housing systems, including outdoor access, natural floor conditions or enrichment material [7,9,10,12]. Furthermore, our findings are similar to those of the study by Kühl et al. [8] who found that citizens clearly rated a free-range system for pigs best and indoor housing with slatted floors worst with regard to animal welfare. In Germany, about 80 percent of pigs are currently kept indoors on fully slatted floors [37]. On average, participants in this study only slightly agreed that most pigs are kept in such housing systems. This reflects participants’ limited knowledge about how farm animals (i.e. pigs) are predominantly kept and could be an indicator that they have even worse pictures in mind, similar to other studies [38,39]. The latter can be supported by our cognitive pre-tests, where some participants stated that ‘indoor’ was still considered a comparatively good housing system and that there are much worse systems, such as keeping pigs in cages, for example. This is also in line with the animal welfare organization’s feedback that our picture selection for this housing system represents a rather positive example of a conventional pig stable. It is conceivable that this may have positively influenced the perception and evaluation in this study, at least to some extent. Nevertheless, this finding makes the very poor acceptability of the indoor system herein even more remarkable.

Looking at the initial acceptability of the alternative housing systems (i.e. before confrontation with trade-off situations), acceptability in both splits increased with the enrichment level of the housing systems. These findings underline citizens’ preferences for the most near-nature and animal-friendly housing conditions. Schütz et al. [40] also demonstrated that citizens correctly recognize at least general differences in the enrichment level (i.e. housing conditions) and therewith the benefit to animal welfare of various housing systems [7,41]. However, looking at the average acceptability levels in split 1, the only alternative housing system that is acceptable for participants is ‘outdoor run’. In contrast, in split 2, all presented alternatives were evaluated as at least rather acceptable. This demonstrates citizens’ willingness to compromise and to accept housing systems that do not correspond to their ideal image but display an improvement to fully slatted indoor housing.

Furthermore, it becomes clear that the type of reference system influenced the evaluation, with a negative reference system leading to a comparatively higher acceptability of alternative housing systems. This influence may be explained by the so-called contrast effect, a cognitive bias phenomenon. Contrast effects result from the use of heuristics in order to better process the vast amount of information we are confronted with every day [42–44]. Contrast effects are ubiquitous and bias our perception of information. Thus, a target stimulus is perceived as higher or lower than its actual value and stands in contrast to a reference stimulus to which we were recently exposed [43,45]. Examples range from sensory stimuli to a wide range of real-life decisions [43]. Thus, a grey square appears darker inside a light grey circle than inside a black circle [43], or a product is perceived more attractive when presented together with less attractive alternatives and vice versa [45]. Against this background, it can be assumed that contrast effects also influenced the results in our study, leading to lower acceptability levels of alternative housing systems in split 1 compared to split 2. This might be relevant when communicating housing conditions of farm animals in general, but also in the context of specific product marketing at the point of sale. Showing a product with a comparatively lower animal welfare level (i.e. indoor housing with slatted floors) might promote a more positive perception of products from more animal-friendly housing systems and thereby potentially increase demand for these products. In this context, an example of a practical implication is the German animal welfare label called ‘Haltungsform’ which comprises four different housing systems ranging from low (i.e. indoor housing = 1) to high animal welfare (i.e. premium = 4) with the latter guaranteeing outdoor access. A picture-based presentation of all four levels would enable consumers to weigh up all alternatives and make a corresponding buying decision. Consequently, higher levels might benefit in the sense that consumers perceive them as even more positive in a direct comparison, which might then also have a positive effect on the sales of these products.

Apart from contrast effects the so-called status quo bias may have also influenced evaluation of the alternative housing systems. The status quo bias describes a cognitive bias where people retain the status quo and avoid change when choosing between alternatives, even if more beneficial options are available [46,47]. This phenomenon is based on loss aversion and regret avoidance with the status quo serving as a reference point, both of which lead people to perceive it as beneficial to maintain the status quo [48]. However, unlike the contrast effect, the status quo bias is rather not suitable to explain the differences in acceptability levels depending on the type of reference system. We rather assume, that status quo bias similarly influenced acceptability in both splits, with participants tending to stick to the reference system (i.e. positive reference system in split 1 and negative in split 2) instead of accepting the offered alternatives. Thus, acceptability levels might have been negatively influenced regardless of the reference system and led to lower acceptability levels in both splits, compared to alternatives being evaluated without reference systems.

### Willingness to compromise in trade-off situations (research question 2)

Within our second research question we wanted to shed light on a) whether citizens’ willingness to compromise (i.e. by accepting alternative housing systems when confronted with advantages/disadvantages) depends on the specific trade-off presented and b) whether it changes through confrontation with trade-off situations.

The extent to which participants are willing to compromise in specific trade-off situations (2a) differs between housing systems, similar to the initial acceptability for these systems (see pervious section). Thus, the more animal-friendly the housing system (i.e. the higher the enrichment level), the more participants in both splits strove to keep compromises at the expense of housing conditions as low as possible by showing higher acceptability rates for all presented trade-off situations (i.e. advantages/disadvantages). However, for all housing systems the willingness to compromise differed among trade-off situations. In split 1, it turned out that the given animal welfare advantage (i.e. animal health) received the highest acceptability and therewith obviously had the strongest persuasive power. This means that compromises in terms of housing system and thus to the expense of animal welfare are most likely to be accepted if this results in an improvement of animal welfare in other welfare domains (i.e. animal health). This may be partly due to the high importance that citizens attribute to animal welfare in general [26,49,50], and is also supported by the findings of Sonntag et al. [11] that identified preferences for animal welfare compared to other aspects in specific trade-off situations. However, other presented advantages, such as those related to human health, environmental or climate protection, had only little less persuasive power than animal health in our study. When presented as a disadvantage (split 2), animal health even had a weaker persuasive power than some other aspects. This might be related to increasing societal concerns about human health and environmental risks in meat production in recent years [20,21] as well as to the fact that animal health on its own might be a comparatively weak argument for animal welfare. The latter is supported by the study of Sonntag et al. [11], where only a minority accepted an increased disease risk in favour of improved housing conditions (i.e. improved thermoregulation, access to fresh air and light, more space), demonstrating a relatively lower prioritization of animal health. Product price was the only aspect that clearly had the least persuasive power compared to the others, both when presented as an advantage as well as a disadvantage. This is also in line with Sonntag et al. [11], where economic trade-offs were neglected over animal welfare trade-offs even though other trade-offs relating to emissions, for example, were on a comparable level. Furthermore, the comparatively high willingness to accept higher product prices in favour of animal welfare in our study corresponds to results from several willingness-to-pay studies that revealed that consumers are willing to pay higher prices for products from more animal-friendly production systems [26,27]. To sum up, according to our results, product price does not seem to be a suitable argument to justify pig housing systems with lower animal welfare levels.

This becomes even clearer when analysing whether the confrontation with trade-off situations changes participants’ willingness to compromise, and whether this effect is only temporary or also sustainable (research question 2b). For this purpose, we first compared the initial acceptability levels of the alternative housing systems with the acceptability levels when confronted with the specific trade-off situations (i.e. temporary effect). Results show that a lower product price presented as an advantage even decreased the acceptability levels in some cases (split 1). Thus, claiming cheap prices as a benefit does not seem to be a suitable approach to convince consumers in their role as citizens of certain housing systems but rather leads to an opposite effect. However, unlike price, in most cases acceptability increased when participants were confronted with advantages, and it decreased compared to the initial acceptability when they were confronted with disadvantages. Thereby, acceptability increased the strongest for the most negative housing system (i.e. ‘indoor’ in split 1) and decreased the strongest for the most positive system (‘free-range’ in split 2). This effect is similar, but much stronger, to the one we found when analysing whether acceptability changed sustainably by comparing the initial with the final acceptability levels (i.e. before and after confrontation with trade-offs). In split 1, this effect could be related to the fact that the more positive a housing system is perceived, the more strongly the system is already associated with benefits, and thus the persuasive power of advantages is reduced (i.e. weakest increase of acceptability). In contrast, the decrease of acceptability in split 2 might be due firstly to a weaker effect of disadvantages compared to advantages and secondly to a potential idealization of the ‘free-range’ housing system. Participants probably associate this housing system primarily with benefits (especially regarding animal welfare), so that the presented disadvantages might have come unexpectedly and, consequently, had a stronger negative effect.

Altogether, our findings demonstrate that the confrontation with either advantages or disadvantages affect the acceptability of alternative housing systems, especially in the short term. This is in contrast to the study of Sonntag et al. [11], which found that information given in form of images and advantages/disadvantages did not increase the acceptance of a system that was initially rated negatively, nor did it decrease the acceptance of a system that was initially rated positively. However, in contrast to Sonntag et al. [11], in our study housing systems were presented as alternatives to either a positive or negative reference system instead of being evaluated separately and thereby included a compromise from the beginning. Furthermore, the confrontation with advantages or disadvantages did not sustainably change the general acceptability level of the presented housing systems in our study either. A particularly negatively perceived housing system (i.e. ‘indoor’) remained unacceptable despite the provided advantages, and a particularly positively perceived housing system (i.e. ‘free-range’ system) remained highly acceptable despite the provided disadvantages. In conclusion, although citizens temporarily adjusted their evaluation in trade-off situations, in the end they were not willing to make huge compromises at the expense of animal welfare by accepting particularly negative housing systems or by rejecting particularly positive ones. According to our results, public desire for high animal welfare levels seems to be relatively stable and is not even shaken by other sustainability arguments, especially not by price.

## Conclusions

Our results confirm that German citizens clearly favour pig housing systems that provide high animal welfare levels, e.g. a free-range system. However, in trade-off situations they become uncertain and adjust their evaluation by giving up on housing conditions in the short term. In this context, the presented trade-offs (i.e. advantages/disadvantages) relating to animal or human health seem to have a stronger persuasive power than those referring to climate protection or product price. However, when it comes to a final evaluation, citizens tend to adhere to their initial evaluation and are not dissuaded from their general desire for high animal welfare. Nevertheless, according to our results, citizens are willing to compromise on pig housing conditions up to a certain level. Thereby, the system ‘indoor housing with straw bedding and outdoor access’ has the potential to be an acceptable compromise to citizens’ ideal view of a housing system (i.e. free-range system). Further, ‘indoor housing with straw bedding’ partially seems to be an option as well. In light of the transformation process of animal husbandry, this means that a change in this direction is needed to be socially acceptable in the long term. From a citizen’s perspective, straw bedding and especially outdoor access are highly important and non-negotiable for animal-friendly pig housing systems. Anything else would not be an acceptable compromise for citizens and would not meet their clear demand for animal welfare–even if advantages of intensive systems or disadvantages of more animal-friendly systems are communicated more strongly. With regard to housing systems with outdoor access for example, which are currently difficult to implement in some parts of Germany due to emissions regulations, according to our results, appropriate framework conditions should be urgently established in favour of animal welfare, even if these may entail disadvantages for the environment, the climate or other areas. In the current debate on agricultural policy possible trade-offs are often used to justify an increasing intensification. However, the high priority citizens attach to animal welfare demonstrates that compromises in this area jeopardize the public acceptance of livestock farming and therewith the ‘social license to produce’ which finally could lead to an increased consumption of meat alternatives or a reduction of consumption of animal products. Thus, in order to comply with societal demands and improve acceptability of animal husbandry, it seems reasonable to consider introducing appropriate animal welfare policies, which comes along with a revision of current animal welfare legislation and the implementation of stricter standards.

## Limitations and future research

The study is subject to some limitations for (online) surveys. Although participants were recruited via panel provider to ensure that the sample’ composition corresponds to the German population at least in terms of some basic sociodemographic characteristics, the so-called selection bias might have influenced the composition of our sample. For example, only people who are literate and have access to the internet were able to participate or those who took part were particularly interested in the survey topic. Moreover, social desirability effects could have played a role in our study, although these are reduced in anonymous online surveys. Furthermore, order effects can influence survey results, which we tried to keep as low as possible, though, by carefully structuring the questionnaire and randomizing the appearance of questions and items.

Even though pictures for our study were selected very carefully and two independent organizations checked up on whether they represent housing systems in a neutral and realistic manner or not, it is still possible that the selection influenced participants’ perceptions and therefore our results. To provide an even more extensive and realistic impression of the housing systems, personal or virtual stable tours (e.g. via Virtual Reality Glasses) could be more suitable, although feasibility would be challenging with large sample sizes. Last but not least, it would have been valuable if we had analyzed the influence of factors such as meat consumption, attitudes towards animal welfare or sociodemographic characteristics including age, gender, education level or household income on citizens’ willingness to compromise in sustainability trade-off situations. Future research could investigate how similar trade-off situations are handled from the consumer’s rather than from the citizen’s point of view (e.g. in a direct buying situation) e.g. by looking into consumers’ willingness to buy or willingness to pay for products coming from certain animal friendly housing systems when confronted with different trade-offs (advantages/disadvantages). Furthermore, future studies could address citizens’ willingness to support policies that aim to improve animal welfare (e.g. by promoting more animal-friendly housing systems, which, indeed, have been shown to meet their preferences). Finally, international studies could be conducted in order to compare citizens’ or consumers’ acceptability of different housing systems against the background of emerging trade-offs.
